# Supplementary material for: Hyperbranched Polylysine Exhibits a Collaborative Enhancement of the Antibiotic Capacity to Kill Gram-Negative Pathogens
Source: Antibiotics (Basel). 2024 Feb 26;13(3):217. doi: 10.3390/antibiotics13030217 (PMC10967504; doi:10.3390/antibiotics13030217)
Supplement: Supplementary file 1 [file antibiotics-13-00217-s001.zip › antibiotics-2808972-supplementary.pdf]

antibiotics

MDPI

Supporting Information

Hyperbranched polylysine exhibits a collaborative enhancement of the antibiotic capacity to kill Gram-negative pathogens

Yuxin Gong [1], Qing Peng [1], Yu Qiao [1], Dandan Tian [1], Yuwei Zhang [2], Xiaoyan Xiong [1], Mengxin He [1], Xiaoqing Xu [1,]* and Bo Shi [1,]*

[1] Feed Research Institute, Chinese Academy of Agricultural Sciences, No. 12 South Zhongguancun Street, Beijing, 100081, China; 82101211095@caas.cn (Y. X. G.); pengqing@caas.cn (Q. P.); qiaoyu@caas.cn (Y. Q.); 82101201074@caas.cn (D. D. T.); 82101215432@caas.cn (X. Y. X.); 82101212159@caas.cn (M. X. H.)

[2] Institute of Agro-Products Preservation and Processing Technology, Tianjin Academy of Agricultural Sciences, Tianjin 300380, China; zhangyuwei2527@163.com

* Correspondence: Authors to whom correspondence should be addressed. xuxiaoqing@caas.cn (X. Q. X.); shibo@caas.cn (B. S.)

Table S1. Fluorescence values obtained in a NPN uptake assay of *S. typhimurium* added with 0.5 and 0.02 mg/mL permeabilization.

Sample	NPN	Fluorescence value	After background	NPN uptake factor
Buffer	−	543.3333333		
Buffer	+	1821.666667	1278.33	1.00
Cell	−	2877.666667		
Cell	+	6788.833333	3911.17	3.06
Cell+HBPL-3 0.5mg/mL	−	8221.833333		
Cell+HBPL-3 0.5mg/mL	+	19247.33333	11025.50	8.62
Cell+HBPL-3 0.02mg/mL	−	3051.666667		
Cell+HBPL-3 0.02mg/mL	+	7393.333333	4341.67	3.40
Cell+HBPL-6 0.5mg/mL	−	38064.83333		
Cell+HBPL-6 0.5mg/mL	+	71002.66667	32937.83	25.77
Cell+HBPL-6 0.02mg/mL	−	5661.833333		
Cell+HBPL-6 0.02mg/mL	+	18151.16667	12489.33	9.77
Cell+HBPL-17 0.5mg/mL	−	76152		
Cell+HBPL-17 0.5mg/mL	+	102447	26295.00	20.57
Cell+HBPL-17 0.02mg/mL	−	3256.666667		
Cell+HBPL-17 0.02mg/mL	+	25229.66667	21973.00	5.62

Table S2. Fluorescence values obtained in a NPN uptake assay of *E. coli* O157：H7 added with 0.5 and 0.02 mg/mL permeabilization.

Sample	NPN	Fluorescence value	After background	NPN uptake factor
Buffer	−	534.3333333		
Buffer	+	1450.5	916.17	1.00
Cell	−	2563		

Cell	+	5410.333333	2847.33	3.11
Cell+HBPL-3 0.5mg/mL	−	7813.5		
Cell+HBPL-3 0.5mg/mL	+	15444.83333	7631.33	8.33
Cell+HBPL-3 0.02mg/mL	−	2713.166667		
Cell+HBPL-3 0.02mg/mL	+	5713.833333	3000.67	3.28
Cell+HBPL-6 0.5mg/mL	−	37423.5		
Cell+HBPL-6 0.5mg/mL	+	61599.16667	24175.67	26.39
Cell+HBPL-6 0.02mg/mL	−	4808.666667		
Cell+HBPL-6 0.02mg/mL	+	14077.33333	9268.67	10.12
Cell+HBPL-17 0.5mg/mL	−	74578.16667		
Cell+HBPL-17 0.5mg/mL	+	94281	19702.83	21.51
Cell+HBPL-17 0.02mg/mL	−	7214.666667		
Cell+HBPL-17 0.02mg/mL	+	18438.83333	11224.17	3.94

Table S3. Fluorescence values obtained in a NPN uptake assay of *P. aeruginosa* PAO1 added with 0.02 mg/mL permeabilization.

Sample	NPN	Fluorescence value	After background	NPN uptake factor
Buffer	−	539.3333333		
Buffer	+	1489	949.67	1.00
Cell	−	3603.333333		
Cell	+	4287	683.67	0.72
Cell+HBPL-3 0.5mg/mL	−	8990.5		
Cell+HBPL-3 0.5mg/mL	+	23654	14663.50	15.44
Cell+HBPL-3 0.02mg/mL	−	3841.833333		
Cell+HBPL-3 0.02mg/mL	+	4670.666667	828.83	0.87
Cell+HBPL-6 0.5mg/mL	−	36080.33333		
Cell+HBPL-6 0.5mg/mL	+	61840.66667	25760.33	27.13
Cell+HBPL-6 0.02mg/mL	−	2838.666667		
Cell+HBPL-6 0.02mg/mL	+	2151.166667	-687.50	-0.72
Cell+HBPL-17 0.5mg/mL	−	76518		
Cell+HBPL-17 0.5mg/mL	+	91367.16667	14849.17	15.64
Cell+HBPL-17 0.02mg/mL	−	8216.833333		
Cell+HBPL-17 0.02mg/mL	+	8739.83333	523.00	0.55

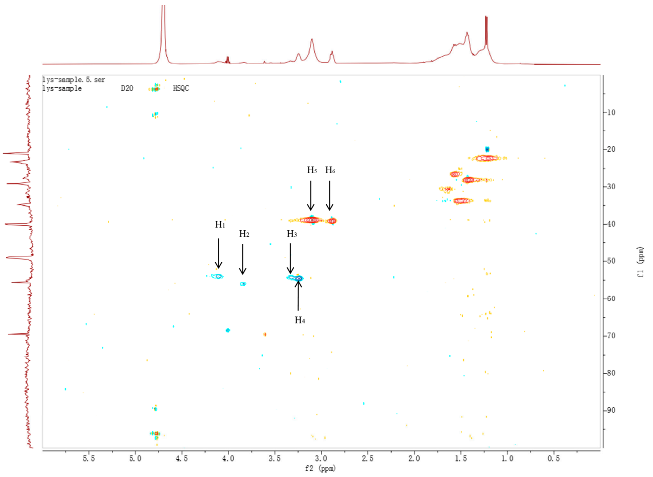

Figure S1. 2D NMR spectra ^{13}C (y axis) ^{1}H (x axis) of HPN in D_2O.

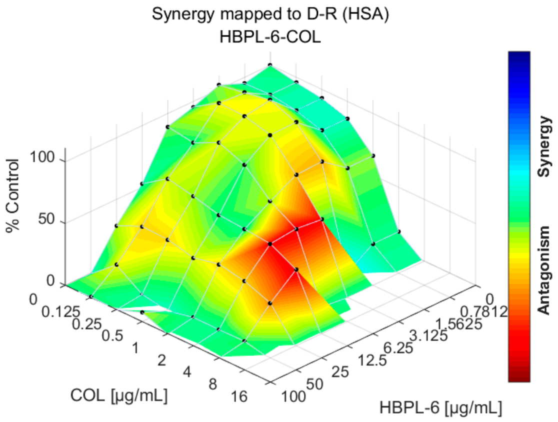

Figure S2. Colistin and ε-PL are antagonistic in treating *S. typhimurium* analysed by Combenefit software.
